# Residential Greenness and Frailty Among Older Adults: A Longitudinal Cohort in China

**DOI:** 10.1016/j.jamda.2019.11.006

**Published:** 2020-06

**Authors:** Anna Zhu, Lijing Yan, Chenkai Wu, John S. Ji

**Affiliations:** a Environmental Research Center, Duke Kunshan University, Kunshan, China; b Global Health Research Center, Duke Kunshan University, Kunshan, China; c Nicholas School of the Environment, Duke University, Durham, North Carolina

**Keywords:** Residential greenness, Normalized Difference Vegetation Index, frailty, healthy longevity

## Abstract

**Objectives:**

Frailty is an accumulation of deficits characterized by reduced resilience to stressors and increased vulnerability to adverse outcomes. There is evolving evidence on the health benefits of residential greenness, but little is known about its impact on frailty.

**Design:**

A longitudinal cohort study.

**Setting and participants:**

We included older adults aged ≥65 years from the Chinese Longitudinal Healthy Longevity Survey (CLHLS) with a 12-year follow-up.

**Methods:**

We assessed residential greenness by calculating the Normalized Difference Vegetation Index (NDVI) in the 500 m radius around participants' residence. We used 39 self-reported health items to construct a frailty index (FI) as a proportion of accumulated deficits. We defined an FI of ≤0.21 as nonfrail and prefrail, and an FI of >0.21 as frail. We used the mixed effects logistic regression models to examine the association between residential greenness and frailty, adjusted for a number of covariates.

**Results:**

We had 16,238 participants, with a mean age of 83.0 years (standard deviation: 11.5). The mean baseline NDVI and FI were 0.40, and 0.12, respectively. Compared to the participants living in the lowest quartile of residential greenness, those in the highest quartile had a 14% [odds ratio (OR): 0.86, 95% confidence interval (CI): 0.77, 0.97] lower odds of frailty. The association was stronger among urban vs rural residents. Additionally, each 0.1-unit increase in annual average NDVI was related to a 2% higher odds of improvement in the frailty status (OR: 1.02, 95% CI: 1.00, 1.04).

**Conclusions and Implications:**

Our study suggests that higher levels of residential greenness are related to a lower likelihood of frailty, specifically in urban areas.

Frailty reflects cumulative physical, psychological, and social deficits with loss reserve and declined resistance to the stressors in the aging process.^1^, ^2^, ^3^ Frailty is related to higher risks of hospitalization,^4^ falls,^5^ and mortality.^6^ It is estimated that about 25% to 50% of adults aged ≥85 years in the world are frail.^7^

There is a lot of research on frailty detection,^7^^,^^8^ the association between frailty and adverse health outcomes,^6^^,^^9^ and individual intervention to prevent and improve frailty.^10^^,^^11^ This research mainly focuses on the individual level. The association between frailty and neighborhood-level factors like the built environment is rarely studied, although evolving evidence showed protective effects of residential greenness on population health, like lower mortality rates and fewer disabilities in activities of daily living.^12^^,^^13^ In Hong Kong, a cohort study of 3240 adults aged 65 years and older showed that higher levels of residential greenness could improve frailty. The association was mediated by physical activity.^14^ Physical activity could be the potential mechanism and was one of the most common interventions to improve frailty.^15^^,^^16^ In addition, frailty may influence access to residential greenness. A study in New Zealand found that the older adults who were the most frail faced a lack of resources to connect with nature.^17^

China has the largest number of older adults aged ≥65 years^18^ and has a large number of older adults who are frail. A study of the 2011-2012 survey of China Health and Retirement Longitudinal Study reported that about 51.2% and 7.0% of older adults aged ≥60 years were prefrail and frail.^19^ The large number of prefrail and frail older adults challenged the long-term care system in China. To our knowledge, the Hong Kong study is the only previous large-scale population-based cohort study on residential greenness and frailty.^14^ However, the association is unclear in the middle-income settings with a greater variation in green space like Mainland China. Our study aimed to examine the association between residential greenness and frailty among Chinese older adults aged 65 years and older.

## Methods

### Study Population

We used 2002, 2005, 2008, and 2011 waves (with a follow-up period between 2002 and 2014) of the Chinese Longitudinal Healthy Longevity Survey (CLHLS). Initiated in 1998, the CLHLS is a prospective longitudinal cohort study on determinants of healthy longevity in China. Follow-up surveys were conducted in 2000, 2002, 2005, 2008, 2011, 2014, and 2018. In each survey, new participants were recruited. The CLHLS used a multistage, stratified cluster design, and randomly selected 631 cities and counties as the sample sites, where the Han Chinese are the largest majority. The sample sites represent about 85% of the Chinese population. The CLHLS recruited a large sample of older adults aged 65 years and older, including both community-dwelling older adults and institutionalized older adults, from 22 of 31 provinces. The CLHLS oversampled the older adults aged ≥80 years, accounting for about 75% of the sample size. Detailed study design and sampling design could be found elsewhere.^20^ The survey has collected extensive data, including demographic characteristic, socioeconomic status (SES), lifestyle, physical and mental health, and health services utilization.^20^ We excluded the 1998 and 2000 waves because all included items used for creating the frailty index (FI) were available since the 2002 survey, and we also excluded the newly recruited participants in the 2014 and 2018 surveys because of unavailable data access.

Among 34,342 participants from the 2002-2011 wave, we excluded the participants if they were missing residential addresses (n = 17), were aged ≤65 years (n = 545), were lost to follow-up (n = 5308), or had died (n = 12,234) after the baseline survey. In total, 16,238 participants were included in the final analysis.

### Greenness Assessment

We calculated the Normalized Difference Vegetation Index (NDVI) to measure the residential greenness. NDVI is a satellite image–based vegetation index that is obtained from the Moderate-Resolution Imaging Spectro-Radiometer (MODIS) in the National Aeronautics and Space Administration's Terra Satellite. In the process of photosynthesis, the plants absorb red visible light and reflect near-infrared light to scatter extra heat. NDVI is equal to the ratio of difference in reflection of visible and near-infrared light to the sum of these 2 measurements. NDVI ranges from −1.0 to 1.0, with larger values indicating higher levels of vegetative density.^21^^,^^22^

Based on the longitude and latitude of residential addresses, we calculated NDVI in the 500 m radius to indicate the residential greenness. Researchers have recommended 0.25 miles (about 400 m) as an optimum radius size to reflect environmental measurement of residential neighbourhood.^23^ A number of similar studies also used the 500 m radius as the scale of greenness exposure around the residency.^24^^,^^25^ MODIS has a temporal resolution of 16 days. We calculated 2 NDVI values for January, April, July, and October from 2002 to 2014 to take into consideration seasonal variation. We calculated annual average NDVI values from 2002 to 2014 to reflect time-varying residential greenness in accordance with multiple measurements of frailty. We categorized annual average NDVI values into quartiles and calculated 0.1 unit of NDVI values.

### Frailty Assessment

We constructed the FI to measure frailty status. Our FI was the same as the previous CLHLS study.^26^ FI included 39 self-reported items, including instrumental activities of daily living, functional limitations, activities of daily living, cognitive function, self-reported health status, interviewer-rated health status, mental health, auditory and visual ability, heart rhythm, and chronic diseases. More details about the included items for calculating the FI can be found in Supplementary Table 1.^26^ We scored each term as 0 (absence of deficit) or 1 (presence of deficit) for 38 of 39 terms. We scored the other 1 term as 2 if the participants reported 2 or more serious illness that caused hospitalization or being bedridden in the past 2 years, such as stroke, cancer, and cataract. FI was equal to the number of reported deficits divided by the total number of included deficits. FI was a continuous variable and ranged from 0 to 1. A higher value indicated poorer frailty.^26^ Our FI reflected cumulative health deficits and was comparable to other studies.^27^^,^^28^ We also classified the continuous FI into nonfrail (FI ≤ 0.21) and frail (FI > 0.21) following a previous report.^29^

### Covariates

We assessed a number of covariates, including age, sex, ethnicity, marital status, geographic region, urban or rural residence, education, occupation, financial support, social and leisure activity, smoking status, drinking status, and physical activity.

### Demographic Characteristics

We calculated the age based on the interview dates and the verified birth dates and divided sex into males and females. We dichotomized ethnicity to Han Chinese and ethnic minorities (Hui, Korean, Manchurian, Mongolian, Yao, Zhuang, and others). We coded marital status as married and not married at the time of interview (separated or divorced, widowed, or never married). Based on the residential addresses of participants, we divided them into 7 geographical regions, including Central China (Henan, Hubei, and Hunan provinces), Eastern China (Anhui, Fujian, Jiangxi, Jiangsu, Shandong, Shanghai, and Zhejiang provinces), Northeastern China (Heilongjiang, Jilin, and Liaoning provinces), Northern China (Hebei, Shanxi, and Tianjin provinces), Northwestern China (Shaanxi Province), Southern China (Guangdong, Guangxi, and Hainan provinces), and Southwestern China (Chongqing and Sichuan provinces).

### Socioeconomic Status

We dichotomized residence to urban or rural areas. The education level of our participants was relatively low. We categorized education into formal education (≥1 year of education) and no formal education (<1 year of education). We divided occupation into professional work (professional and technical personnel, government, and management) and nonprofessional work (agriculture, fishing, service, industry, and housework). We dichotomized financial support into financial independence, where participants were financially independent with their work and retirement wage, and financial dependence, where participants financially relied on other family members.

### Health Behavior

We assessed social and leisure activity index by 7 activities, including gardening, personal outdoor activities excluding exercise, raising poultry or pets, reading, playing cards or mah-jongg, listening to the radio or watching TV, and participating in organized social activities. We scored each activity as 0 (no) or 1 (yes). The index ranged from 0 to 7, with higher values indicating more active social and leisure activity.^30^ We evaluated smoking status by the questions of “whether smoke at present,” and “whether smoked in the past.” We defined never smokers as those who neither smoked in the past nor at the time of interview, former smokers as those who smoked in the past but not at the time of interview, and current smokers as those who smoked at the time of interview. We defined never, former, and current drinkers using a similar evaluation. We evaluated physical activity by the question of “exercise or not at present.”

### Statistical Analysis

We used the mixed effects logistic regression models to assess the association between annual average NDVI (both in quartiles and 0.1 unit of NDVI) and frailty (nonfrail and prefrail vs frail), adjusted for length of follow-up, age, sex, ethnicity, marital status, geographic region, urban or rural residence, education, occupation, financial support, social and leisure activity, smoking status, drinking status, and physical activity. Annual average NDVI was repeatedly measured, based on the updated residential addresses if there was any change in residence during the follow-up period. We calculated odds ratios (ORs) and 95% confidence intervals (CIs) to estimate the odds of being frail under different levels of residential greenness. We plotted cubic splines with 3 knots to visualize the curve association. In addition, we conducted a sensitivity analysis on annual average NDVI and FI at baseline using the logistic regression and mixed effects logistic regression for all participants with or without follow-up surveys. Furthermore, we conducted stratified analysis by age groups, sex, urban or rural residence, occupation (professional work vs nonprofessional work), education (formal education vs no formal education), financial support (financial independence vs financial dependence), social and leisure activity (less active vs more active), smoking status (never vs former vs current smokers), drinking status (never vs former vs current drinkers), and physical activity (yes vs no).

We used the mixed effects ordered logistic regression models to examine the relationship between annual average NDVI at time T – 1 and frailty transitions between time T – 1 and time T among the participants with follow-up surveys. We defined frailty transitions between time T – 1 and time T as deteriorated, stable, and improved in frailty status, using a definition similar to the one in the Hong Kong study.^14^ We defined deterioration in frailty status relative to the reference group. The regression models were adjusted for age, sex, ethnicity, marital status, geographic region, urban or rural residence, education, occupation, financial support, social and leisure activity, smoking status, drinking status, and physical activity.

We reported results of age-adjusted models and fully adjusted models, and results for quartiles of NDVI and 0.1 unit of NDVI. Missing values accounted for less than 2% of the total sample size; we assigned modal and mean values to the missing data for the categorical and continuous variables. We used STATA 14.0 (StataCorp LP, College Station, TX) for statistical analysis.

### Ethical Approval

The CLHLS study was approved by the relevant Institutional Review Board. All participants signed written informed consent.

## Results

Table 1 presents the baseline characteristics of CLHLS participants. We included a total of 16,238 participants. The mean age was 83.0 years [standard deviation (SD): 11.5], 60.9% were 80 years and older, 44.4% were male, and 79.9% lived in rural areas. Urban residents lived in less green areas, had higher SES, and were more physically active than their rural counterparts (Supplementary Table 2). The mean baseline NDVI and FI were 0.40 (SD: 0.15) and 0.12 (SD: 0.12), respectively. Baseline annual average NDVI values were much higher in rural areas than in urban areas (0.44 vs 0.23, *P* < .001). Centenarians (n = 1978) and female participants (n = 9023) had worse frailty. In addition, compared with the participants with follow-up surveys, those without follow-up surveys were older (92 vs 83 years) and more frail (0.24 vs 0.12) at baseline (Supplementary Table 3).

**Table 1:** Baseline Characteristics of CLHLS Participants

Characteristics	Total, n (%)	Baseline Annual Average NDVI	*P* value	Frailty Index	*P* value
	16,238	0.40 ± 0.14	—	0.12 ± 0.12	—
Age, y, mean ± SD	83 ± 11.5	—	—	—	—
Age group, y			<.001		<.001
65-79	6343 (39.1)	0.39 ± 0.15		0.07 ± 0.07	
80-89	4665 (28.7)	0.40 ± 0.14		0.11 ± 0.11	
90-99	3252 (20.0)	0.40 ± 0.14		0.16 ± 0.13	
≥100	1978 (12.2)	0.41 ± 0.14		0.24 ± 0.16	
Sex			.014		<.001
Male	7215 (44.4)	0.39 ± 0.14		0.09 ± 0.10	
Female	9023 (55.6)	0.40 ± 0.14		0.14 ± 0.13	
Ethnicity			<.001		<.001
Han Chinese	15,226 (93.8)	0.39 ± 0.14		0.12 ± 0.12	
Ethnic minority	1012 (6.2)	0.47 ± 0.13		0.10 ± 0.11	
Marital status			<.001		<.001
Married	6269 (38.6)	0.39 ± 0.15		0.08 ± 0.09	
Not married	9969 (61.4)	0.40 ± 0.14		0.14 ± 0.13	
Residence			<.001		<.001
Urban area	3266 (20.1)	0.23 ± 0.12		0.13 ± 0.13	
Rural area	12,972 (79.9)	0.44 ± 0.12		0.12 ± 0.12	
Occupation			<.001		<.001
Professional work	1316 (8.1)	0.31 ± 0.15		0.09 ± 0.11	
Nonprofessional work	14,922 (91.9)	0.40 ± 0.14		0.12 ± 0.12	
Education			<.001		<.001
Formal education	6569 (40.5)	0.37 ± 0.15		0.09 ± 0.10	
No formal education	9669 (59.5)	0.41 ± 0.14		0.14 ± 0.13	
Financial support			<.001		<.001
Financial independence	5044 (31.1)	0.35 ± 0.16		0.08 ± 0.09	
Financial dependence	11,194 (68.9)	0.42 ± 0.13		0.14 ± 0.13	
Social and leisure activity index, mean ± SD	2.49 ± 1.53	—	—	—	—
Smoking status			<.001		<.001
Never smoker	10,678 (65.8)	0.40 ± 0.14		0.13 ± 0.13	
Former smoker	2190 (13.5)	0.37 ± 0.14		0.12 ± 0.12	
Current smoker	3370 (20.7)	0.40 ± 0.14		0.08 ± 0.09	
Drinking status			<.001		<.001
Never drinker	11,131 (68.5)	0.39 ± 0.15		0.13 ± 0.13	
Former drinker	1571 (9.7)	0.39 ± 0.14		0.13 ± 0.13	
Current drinker	3536 (21.8)	0.41 ± 0.14		0.09 ± 0.10	
Physical activity			<.001		<.001
Yes	5313 (32.7)	0.35 ± 0.15		0.08 ± 0.08	
No	10,925 (67.3)	0.42 ± 0.13		0.14 ± 0.13	
Geographic region			<.001		<.001
Central China	2474 (15.2)	0.44 ± 0.12		0.12 ± 0.12	
Eastern China	6327 (40.0)	0.40 ± 0.15		0.12 ± 0.12	
Northeastern China	1278 (7.9)	0.27 ± 0.11		0.14 ± 0.14	
Northern China	742 (4.6)	0.26 ± 0.11		0.13 ± 0.13	
Northwestern China	216 (1.3)	0.37 ± 0.13		0.13 ± 0.12	
Southern China	3144 (19.4)	0.44 ± 0.13		0.10 ± 0.11	
Southwestern China	2057 (12.7)	0.39 ± 0.13		0.12 ± 0.12	

Data are mean ± SD for baseline annual average NDVI and frailty index.

**Fig. 1:**
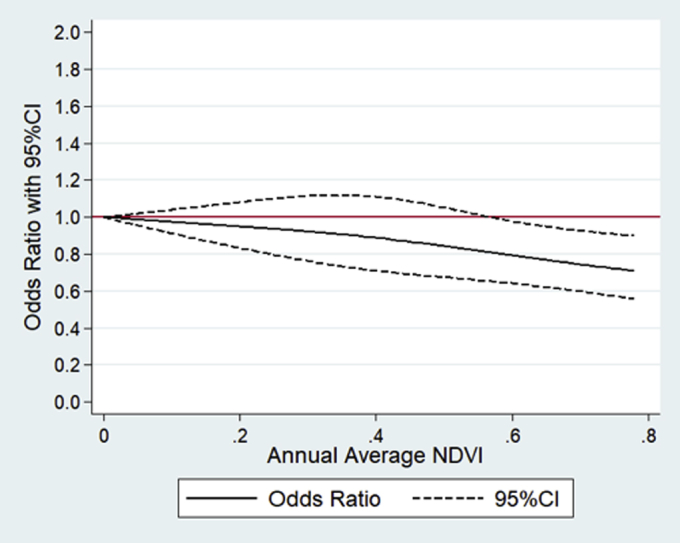
Curve association between annual average NDVI and frailty. Data are shown as OR (95% CI) of being frail in the fully adjusted models.

Table 2 reports the results of mixed effects logistic regression for the association between annual average NDVI and frailty. In the fully adjusted regression, each 0.1-unit increase in annual average NDVI was associated with an OR of 0.96 (95% CI: 0.93, 0.99) of frailty. Compared with the participants living in the lowest quartile of residential greenness, those in the highest quartile had a 14% lower odds of frailty (OR: 0.86, 95% CI: 0.77, 0.97). It has an equivalent effect of 1-year increase in age on frailty (OR: 1.14, 95% CI: 1.13, 1.14). The cubic splines (Figure 1) reported consistent findings that higher levels of annual average NDVI were associated with lower odds of frailty. Additionally, the sensitivity analysis including the participants both with and without follow-up surveys by using a cross-sectional design reported a similar protective association between annual average NDVI and frailty (Supplementary Tables 4 and 5). Furthermore, the association was only significant among the urban residents (OR: 0.89, 95% CI: 0.82, 0.96), not rural residents (OR: 0.98, 95% CI: 0.95, 1.02).

**Table 2:** Mixed Effects Logistic Regression for the Relationship Between Annual Average NDVI and Frailty

Exposure Metrics	Age-Adjusted OR (95% CI)	Fully Adjusted∗ OR (95% CI)
All participants (N = 16,238)		
Quartiles of NDVI		
Quartile 1	Ref	Ref
Quartile 2	0.78 (0.71, 0.84)	0.92 (0.83, 1.03)
Quartile 3	0.71 (0.65, 0.78)	0.94 (0.84, 1.06)
Quartile 4	0.62 (0.57, 0.68)	0.86 (0.77, 0.97)
0.1-unit of NDVI	0.93 (0.91, 0.95)	0.96 (0.93, 0.99)
Urban area (n = 3266)		
Quartiles of NDVI		
Quartile 1	Ref	Ref
Quartile 2	0.80 (0.66, 0.96)	0.76 (0.61, 0.96)
Quartile 3	0.70 (0.52, 0.94)	0.71 (0.50, 1.01)
Quartile 4	0.73 (0.51, 1.04)	0.66 (0.43, 1.01)
0.1-unit of NDVI	0.96 (0.90, 1.02)	0.89 (0.82, 0.96)
Rural area (n = 12,972)		
Quartiles of NDVI		
Quartile 1	Ref	Ref
Quartile 2	0.92 (0.82, 1.02)	1.01 (0.89, 1.15)
Quartile 3	0.86 (0.77, 0.97)	1.04 (0.91, 1.19)
Quartile 4	0.75 (0.67, 0.84)	0.96 (0.84, 1.09)
0.1-unit of NDVI	0.99 (0.97, 1.02)	0.98 (0.95, 1.02)

ORs (95% CIs) of being frail are shown.

∗ In the fully adjusted models, ORs were adjusted for length of follow-up, age, sex, ethnicity, marital status, geographic region, urban or rural residence, education, occupation, financial support, social and leisure activity, smoking status, drinking status, and physical activity.

**Fig. 2:**
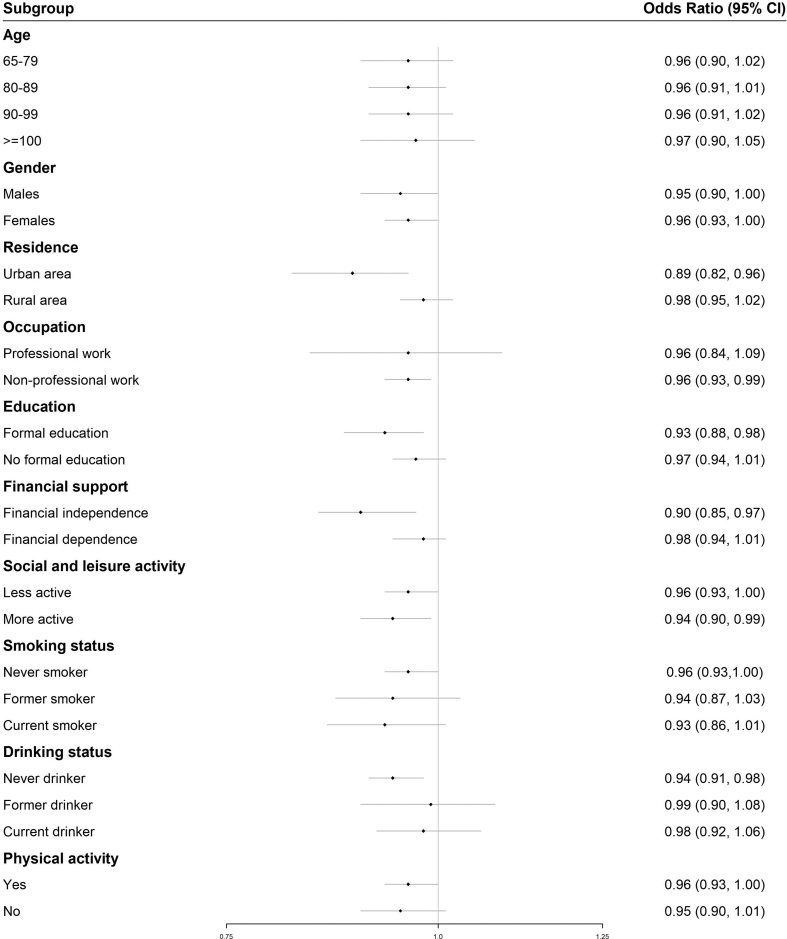
Stratified analysis for each 0.1-unit increase in annual average NDVI and frailty. Data are shown as OR (95% CI) of being frail in the fully adjusted models.

Figure 2 shows the stratified analysis on each 0.1-unit increase in annual average NDVI and frailty. We observed significant association only among the participants who were living in urban areas, had nonprofessional work, had formal education, were financially independent, and were never drinkers, compared with their counterparts.

Table 3 shows the association between annual average NDVI and frailty transitions. In the fully adjusted regression, each 0.1-unit increase in annual average NDVI was related to a 2% higher odds of improvement in the frailty status (OR: 1.02, 95% CI: 1.00, 1.04), and the association was weak but detectable.

**Table 3:** Mixed Effects Ordered Logistic Regression for the Association Between Annual Average NDVI and Frailty Transitions

Exposure Metrics	Age-Adjusted OR (95% CI)	Fully Adjusted∗ OR (95% CI)
Quartiles of NDVI		
Quartile 1	Ref	Ref
Quartile 2	1.08 (1.01, 1.15)	1.08 (1.00, 1.16)
Quartile 3	1.11 (1.04, 1.18)	1.09 (1.02, 1.18)
Quartile 4	1.07 (1.00, 1.14)	1.06 (0.98, 1.14)
0.1-unit of NDVI	1.02 (1.01, 1.04)	1.02 (1.00, 1.04)

ORs (95% CIs) of improvement in frailty status are shown, which have deteriorated in frailty status as the reference group.

∗ In the fully adjusted models, ORs were adjusted for age, sex, ethnicity, marital status, geographic region, urban or rural residence, education, occupation, financial support, social and leisure activity, smoking status, drinking status, and physical activity.

## Discussion

We found that living in the highest quartile of residential greenness was associated with a 14% lower odds of frailty, compared with the lowest quartile. Together with our previous study that higher levels of residential greenness were associated with lower mortality rates and fewer disabilities in activities of daily living,^12^^,^^13^ our findings suggested the potential health benefits of residential greenness, which have significant medical implications. Frailty influences multiple physiologic systems when reaching an aggregate threshold of declined resistance to the stressors. Patients who are frail have specific care needs on progressive weakness, weight loss, decreased exercise tolerance, and slowed task performance.^2^ They also have lower abilities to tolerate the medical procedures and hospitalization.^2^ Increasing green space and appropriate planning around the residency, especially for the nursing homes, may reduce specific care needs and improve the effectiveness of relevant treatment, which requires further study.

Our finding was consistent with the other study in Hong Kong. The Mr. and Ms. Os (Hong Kong) Study, composed of 3240 older adults aged ≥65 years, reported that compared with the lowest quartile, those living in the highest quartile of green space at baseline in the 300 m radius around the residence were related to a 29% higher odds of improving frailty, which was evaluated by 5-item Cardiovascular Health Study frailty phenotype.^14^ To our knowledge, there are no other large-scale population-based cohort studies on residential greenness and frailty. Hong Kong is the most populous and developed place in the world whereas our participants were from 22 of 31 provinces in Mainland China, and up to 79.9% were rural residents. Our study added additional evidence in developing contexts with a greater variation in residential greenness.

Our study found that there was a difference in the association between residential greenness and frailty by urban and rural residence. The association was only significant for the urban residents, rather than those living in rural areas where there was more green space. Evidence on the urban-rural difference in the association between green space and health outcome was not consistent among other studies. A study of 250,782 participants in the Netherlands reported the significant association between green space and perceived general health among all degrees of urbanity (from strongly urban to nonurban areas), although the effect size varied.^31^ The authors found that the urban-rural difference could be explained by the amount of green space, which had stronger effects on health than the degree of urbanity. Another study in England showed that the relationship between green space and health depended on both levels of urbanity and income deprivation. There was no significant association in higher-income suburban and higher-income rural areas, whereas more green space was related to worse health in lower-income suburban areas.^32^ It was possible that there were different types of vegetation between higher-SES and lower-SES communities. It is unclear how different types of vegetation influences population health. Overall, few studies reported the urban-rural difference in the association between green space and health outcomes because majority studies were in the developed contexts and mainly focus on urban areas. Additionally, the urban-rural difference in our study might also be due to the urban-rural difference in frailty. Our study showed that the urban residents were a little bit frailer than the rural residents (0.13 vs 0.12 for FI at baseline). Furthermore, the difference might be explained by the urban-rural difference in the structure of green space and types of vegetation, which might have different effects on frailty.^33^ A study consisting of 6 million urban residents in Taipei showed that maximizing the largest green patch proportion and minimizing green space fragmentation were the most important contributors to reduce cardiovascular mortality.^34^ A study involving 276 counties in the United States reported similar findings that the effects of green space on mental effects also differed by the structures of green space.^35^ The structure of green space and types of vegetation may be different in urban and rural area in China. We did not have relevant data, and we could not verify the potential hypothesis. Further research on comparing the structure of green space and types of vegetation between urban and rural areas and their health effects is needed.

We did not observe significant gender difference in the association between residential greenness and frailty. This is not consistent with the Mr. and Ms. Os Study in Hong Kong,^14^ which reported health benefits of residential greenness on frailty only among males but not females. Gender difference was also reported in another study in the United Kingdom, which stated that males and females may have different perceptions and usage of green space.^36^ Our study did not have information on the usage pattern of green space by gender. It was unclear how the usage pattern could influence the association by gender.

There are several limitations to our study. First, although NDVI could objectively indicate the density of overall vegetation, it could not indicate the structure of green space and specific types of vegetation. The activity patterns of participants were also unknown. It was unclear how different types of vegetation and activity patterns could influence the association between residential greenness and frailty. Our study only used the 500 m radius around the residential address. It was unknown whether other scales had stronger or weaker associations. Second, our study had a potential concern about informative censoring because of our longitudinal design. Partly because of the advanced age (mean age: 83 years) of our participants, there were a large number of deaths (n = 12,234) and many were lost to follow-up (n = 5308) after the baseline survey. Compared with all participants at baseline, the participants with follow-up surveys were much younger (83 vs 88 years) and had better frailty (0.12 vs 0.18) at baseline (Supplementary Table 3). However, our sensitivity analysis on residential greenness and frailty included all participants with or without follow-up surveys, which used a cross-sectional design, and showed consistent findings with the longitudinal analysis (Supplementary Tables 4 and 5). These consistent findings confirmed the protective effects of residential greenness on frailty, and made our evidence more convincing. Although the cross-sectional design had some limitations, it could help decrease the potential selection bias and dropout bias from the longitudinal analysis, and also contributed to a more comprehensive picture. Third, although our FI was a continuous variable, we categorized it into frailty categories (nonfrail, prefrail, and frail). It made FI less sensitive because continuous FI was more informative to describe the deficit accumulation and the health status trajectories. The continuous FI was also less likely to misclassify phenotype.^37^ However, the cutoff points used in our study have been illustrated to be reliable in the other study.^29^ Categorizing FI in our study shall not bias the association. Fourth, our study lacks evidence on mechanisms of residential greenness and frailty. The path analysis in the Hong Kong study illustrated that residential greenness could directly, and indirectly, influence frailty through physical activity.^14^ However, our study did not show different associations by physical activity. We also found that the association between residential greenness and frailty became nonsignificant if social and leisure activity and physical activity were not adjusted. It was possible that residential greenness was more likely to directly influence frailty in our study, or our measurement of physical activity was not accurately measured by the question of “exercise or not at present.” While there might be mediating effects of physical activity, we could not conduct path analysis like the Hong Kong study because we lacked more accurate and detailed information to define the mediators like physical activity, and our FI assessment included potential mediators, such as cognitive function and mental health. Lastly, compared with the socioeconomic characteristics defined in other studies in developed contexts, our definitions may appear to be relatively crude. However, they reflected the special contexts in China of our participants' generation. With the mean age of 83 years at the time of the interview, our participants were mainly born between the 1900s and 1940s, when China experienced unstable and disrupted economic activities. Although our definitions of personal characteristics might not eliminate all residual confounding, they were a good proxy.

Our study has several strengths. Our study added evidence on residential greenness and frailty in the developing context, and provided evidence for further research on preventing or delaying frailty. Consistent with our previous analysis on residential greenness and mortality,^12^ our findings further reinforced the evidence on the potential health benefits of residential greenness. Additionally, our study included a large sample of 16,238 participants, recruited from 22 of 31 provinces in Mainland, China. This unique sampling design provided evidence in the developing context with a greater variation in residential greenness. Furthermore, our study used a longitudinal design with a long follow-up period from 2002 to 2014. Finally, we assessed greenness exposure by calculating NDVI values, based on aerial and satellite images. We also calculated time-varying annual average NDVI values in accordance with multiple measurements of frailty. This more objective and multiple measurements made our evidence on the residential greenness and frailty more convincing.

## Conclusions and Implications

Our study found that higher levels of residential greenness were associated with a lower likelihood of frailty among Chinese older adults. We observed a stronger association among the urban residents than the rural residents. Our finding reinforced the evidence on the potential health benefit of residential greenness, which had significant implications for supporting the usage of green space to help prevent or improve frailty among Chinese older adults in the process of urbanization and aging.

## Figures and Tables

**Supplementary Table 4:** A Cross-Sectional Analysis for Baseline Annual Average NDVI and Frailty at Baseline (N = 33,780)

Exposure Metrics	Baseline NDVI, Mean ± SD	Baseline FI, Mean ± SD	Age-Adjusted OR (95% CI)	Fully Adjusted∗ OR (95% CI)
Quartiles of NDVI				
Quartile 1	0.18 ± 0.06	0.19 ± 0.18	Ref	Ref
Quartile 2	0.36 ± 0.04	0.18 ± 0.17	0.79 (0.73, 0.85)	0.87 (0.79, 0.96)
Quartile 3	0.46 ± 0.02	0.18 ± 0.16	0.76 (0.71, 0.82)	0.88 (0.80, 0.98)
Quartile 4	0.56 ± 0.04	0.17 ± 0.16	0.67 (0.62, 0.72)	0.81 (0.73, 0.90)
0.1-unit of NDVI	—	—	0.90 (0.89, 0.92)	0.95 (0.92, 0.97)

ORs (95% CIs) of being frail are shown.

∗ In the fully adjusted regression models, ORs were adjusted for age, sex, ethnicity, marital status, geographic region, urban or rural residence, education, occupation, financial support, social and leisure activity, smoking status, drinking status, and physical activity.

**Supplementary Table 3:** Baseline Annual Average NDVI, and Frailty for the Participants With or Without Follow-up Surveys

	All Participants (N = 33,780)	Participants With Follow-up Surveys (n = 16,238)	Participants Without Follow-up Surveys		
			All (n = 17,542)	Death (n = 12,234)	Lost to Follow-up (n = 5308)
Baseline NDVI, mean ± SD	0.39 ± 0.15	0.40 ± 0.14	0.38 ± 0.15	0.40 ± 0.14	0.35 ± 0.16
Baseline FI score, mean ± SD	0.18 ± 0.17	0.12 ± 0.12	0.24 ± 0.18	0.26 ± 0.18	0.19 ± 0.18
Age, mean ± SD	88 ± 11.8	83 ± 11.5	92 ± 10.2	94 ± 8.5	87 ± 12.1

**Supplementary Table 5:** Mixed Effects Logistic Regression for the Relationship Between Annual Average NDVI and Frailty

Exposure Metrics	Age-Adjusted OR (95% CI)	Fully Adjusted∗ OR (95% CI)
All participants (N = 33,780)		
Quartiles of NDVI		
Quartile 1	Ref	Ref
Quartile 2	0.75 (0.69, 0.82)	0.89 (0.81, 0.97)
Quartile 3	0.68 (0.62, 0.74)	0.87 (0.79, 0.96)
Quartile 4	0.59 (0.54, 0.64)	0.81 (0.73, 0.89)
0.1-unit of NDVI	0.87 (0.85, 0.89)	0.94 (0.92, 0.96)
Urban area (n = 7649)		
Quartiles of NDVI		
Quartile 1	Ref	Ref
Quartile 2	0.72 (0.60, 0.86)	0.70 (0.59, 0.84)
Quartile 3	0.70 (0.52, 0.93)	0.68 (0.51, 0.90)
Quartile 4	0.61 (0.43, 0.88)	0.56 (0.39, 0.79)
0.1-unit of NDVI	0.88 (0.83, 0.94)	0.88 (0.83, 0.93)
Rural area (n = 26,131)		
Quartiles of NDVI		
Quartile 1	Ref	Ref
Quartile 2	1.06 (0.95, 1.18)	1.01 (0.91, 1.13)
Quartile 3	0.99 (0.89, 1.10)	0.99 (0.89, 1.11)
Quartile 4	0.87 (0.78, 0.97)	0.93 (0.83, 1.04)
0.1-unit of NDVI	0.94 (0.92, 0.97)	0.97 (0.94, 0.99)

ORs (95% CIs) of being frail are shown.

∗ In the fully adjusted regression models, ORs were adjusted for age, sex, ethnicity, marital status, geographic region, urban or rural residence, education, occupation, financial support, social and leisure activity, smoking and drinking status, and physical activity.

**Supplementary Table 2:** Baseline Characteristics by Urban or Rural Residence Among the Participants With Follow-up Surveys (n = 16,238)

Characteristics	Urban Area	Rural Area
	3266	12,972
Age, y, mean ± SD	83 ± 11.7	83 ± 11.5
Baseline NDVI, mean ± SD	0.23 ± 0.12	0.44 ± 0.12
Baseline FI, mean ± SD	0.13 ± 0.13	0.12 ± 0.12
Sex		
Male	1465 (44.9)	5750 (44.3)
Female	1801 (55.1)	7222 (55.7)
Ethnicity		
Han Chinese	3168 (97.0)	12,058 (92.9)
Ethnic minority	98 (3.0)	914 (7.1)
Marital status		
Married	1358 (41.6)	4911 (37.9)
Not married	1908 (58.4)	8061 (62.1)
Occupation		
Professional work	692 (21.2)	624 (4.8)
Nonprofessional work	2574 (78.8)	12,348 (95.2)
Education		
Formal education	1845 (56.5)	4724 (36.4)
No formal education	1421 (43.5)	8248 (63.6)
Financial support		
Financial independence	1905 (58.3)	3139 (24.2)
Financial dependence	1361 (41.7)	9833 (75.8)
Social and leisure activity index (mean ± SD)	2.97 ± 1.60	2.37 ± 1.49
Smoking status		
Never smoker	2093 (64.1)	8585 (66.2)
Former smoker	602 (18.4)	1588 (12.2)
Current smoker	571 (17.5)	2799 (21.6)
Drinking status		
Never drinker	2314 (70.9)	8817 (68.0)
Former drinker	351 (10.7)	1220 (9.4)
Current drinker	601 (18.4)	2935 (22.6)
Physical activity		
Yes	1815 (55.6)	3498 (27.0)
No	1451 (44.4)	9474 (73.0)
Geographic region		
Central China	297 (9.1)	2177 (16.8)
Eastern China	1150 (35.2)	5177 (39.9)
Northeastern China	689 (21.1)	589 (4.5)
Northern China	425 (13.0)	317 (2.4)
Northwestern China	52 (1.6)	164 (1.3)
Southern China	241 (7.4)	2903 (22.4)
Southwestern China	412 (12.6)	1645 (12.7)

**Supplementary Table 1:** The List of Items Included in Calculating the FI

No.	Items
1	IADL: Unable to visit neighbors by oneself
2	IADL: Unable to shop by oneself if necessary
3	IADL: Unable to cook meals by oneself if necessary
4	IADL: Unable to wash clothing by oneself
5	IADL: Unable to walk continuously for 1 km
6	IADL: Unable to lift a weight of 5 kg (such as a heavy bag of groceries)
7	IADL: Unable to continuously crouch and stand up 3 times
8	IADL: Unable to use public transportation
9	Functional limitations: Unable to put hand behind neck
10	Functional limitations: Unable to put hand behind lower back
11	Functional limitations: Unable to raise arm upright
12	Functional limitations: Unable to stand up from sitting in a chair
13	Functional limitations: Unable to pick up a book from the floor
14	ADL: Needs assistance bathing
15	ADL: Needs assistance dressing
16	ADL: Needs assistance toileting
17	ADL: Needs assistance in indoor transferring
18	ADL: Needs assistance eating
19	ADL: Incontinence
20	Cognitively impaired (based on the MMSE)
21	Poor self-rated health
22	Health worsened in the past year
23	Poor interviewer-rated health
24	Hearing loss
25	Vision loss
26	Abnormal heart rhythm
27	Symptom of psychological distress (based on loneliness, usefulness, fearfulness)
28	Number of serious illnesses in the past 2 years
29	Suffering from hypertension
30	Suffering from diabetes
31	Suffering from tuberculosis
32	Suffering from heart disease
33	Suffering from stroke/cerebrovascular disease
34	Suffering from bronchitis, emphysema, asthma, or pneumonia
35	Suffering from cancer
36	Suffering from arthritis
37	Suffering from bedsores
38	Suffering from gastric or duodenal ulcers
39	Suffering from Parkinson's disease

IADLs, instrumental activities of daily living; ADL, activities of daily living.

Item no. 28 was assigned a value of 2.
